# Robot locomotion by fluid–fluid interaction

**DOI:** 10.1038/s41598-023-49193-z

**Published:** 2023-12-11

**Authors:** Hiroto Kitamori, Shunsuke Kudoh, Jun Shintake

**Affiliations:** Department of Mechanical and Intelligent Systems Engineering, The University of Electro-Communications, 1-5-1 Chofugaoka, Chofu, 182-8585 Tokyo Japan

**Keywords:** Engineering, Materials science

## Abstract

This paper describes a locomotion strategy for robots based on the interaction between two fluids, through the development of an untethered mobile robot. The fundamental principle of robot locomotion is to exploit the active deformations of ferrofluid caused by internal magnetic fields, which generate reaction forces to the surrounding fluid (in this study, water). The developed robot is equipped with two permanent magnets (PMs), two electromagnets (EMs), two clusters of ferrofluid, and a control unit with batteries. It has a length, width, and mass of 107 mm, 94 mm, and 127 g, respectively. In the robot, PMs are used to hold clusters of ferrofluid. The activation of EMs by the controller achieves forward and rotational movements of the robot. Experimental results show the forward speed and rotational speed in water to be 2.7 mm/s (at a driving frequency of 9 Hz) and 1.2°/s (at a driving frequency of 7 Hz), respectively. The measured thrust force of the robot is 2 mN, further supporting the concept of robot locomotion by fluid–fluid interaction.

## Introduction

Robots locomote based on the physical interaction between their body and the surrounding environment. Normally, the bodies of robots are solid; therefore, the majority of robotic locomotion on Earth is explicated as solid–solid (e.g., ground) and solid–fluid (e.g., air and water) interaction. The former is employed in, for instance, legged robots that move via interaction between the legs and the ground (this assumes the presence of a gravitational field capable of generating sufficient frictional force). The latter includes flying robots and swimming robots that locomote through the interaction between their body and the surrounding fluid.

**Figure 1:**
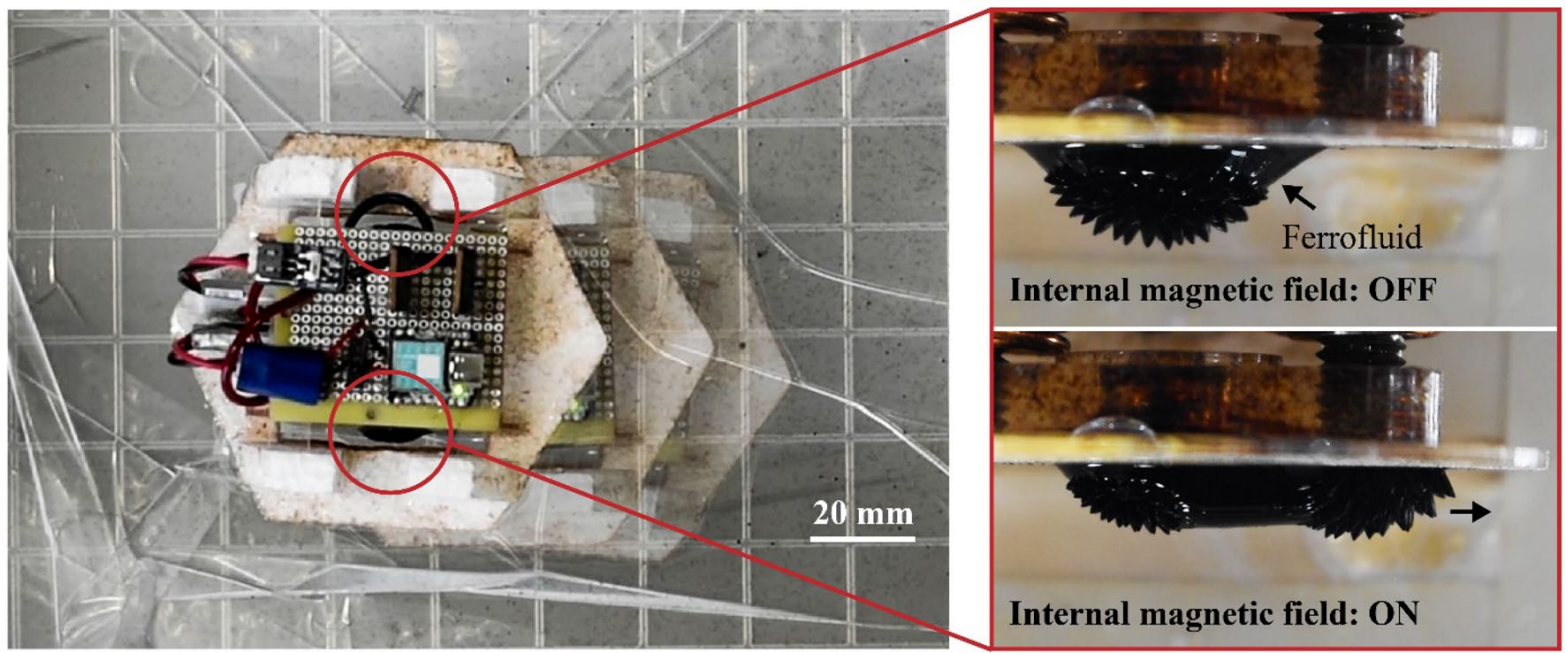
Movement of the robot on the surface of water. The robot is equipped with two clusters of ferrofluid that are immersed in water. Activation of the internal electromagnets generates magnetic fields and induces a rapid shape change of the ferrofluid, which pushes the surrounding water and results in the movement of the robot in a specific direction (in the case of this figure, a forward movement from right to left).

Here, we present a robot that can locomote by fluid–fluid interaction. The idea is to use ferrofluid as the body of the robot, which can be actively deformed by magnetic fields. When the robot is surrounded by an immiscible fluid, the deformations of ferrofluid generate movement, as shown in Fig. 1.

Our robots may be classified as liquid robots^1^. Researchers have demonstrated movement and manipulation of a liquid cluster based on ferrofluids^2–4^ and liquid metals^5–9^. Furthermore, ferrofluids have also been reported to enable in-memory computation^10^. These robotic systems usually move by applying external electromagnetic fields. Moreover, their movements are the result of a perfect balance between the surface tension of the fluid, the static electromagnetic field, magnetization, including that caused by the fluid itself, and the strength of the externally applied field. An exceptional work presented in Ref.^11^ demonstrates ground locomotion (i.e., fluid–solid interaction) using liquid metal. Our robot essentially differs from them in the sense that it locomotes using an internal magnetic field and reaction forces resulted from fluid–fluid interaction.

In this study, we focus on investigating the locomotion characteristics derived by fluid–fluid interaction through characterization of the robot. Specifically, we measure the forward speed, rotational speed, and thrust force generated by fluid–fluid interaction as functions of the switching frequency of the internally generated magnetic field and discuss on the results.

## Results and discussion

**Figure 2:**
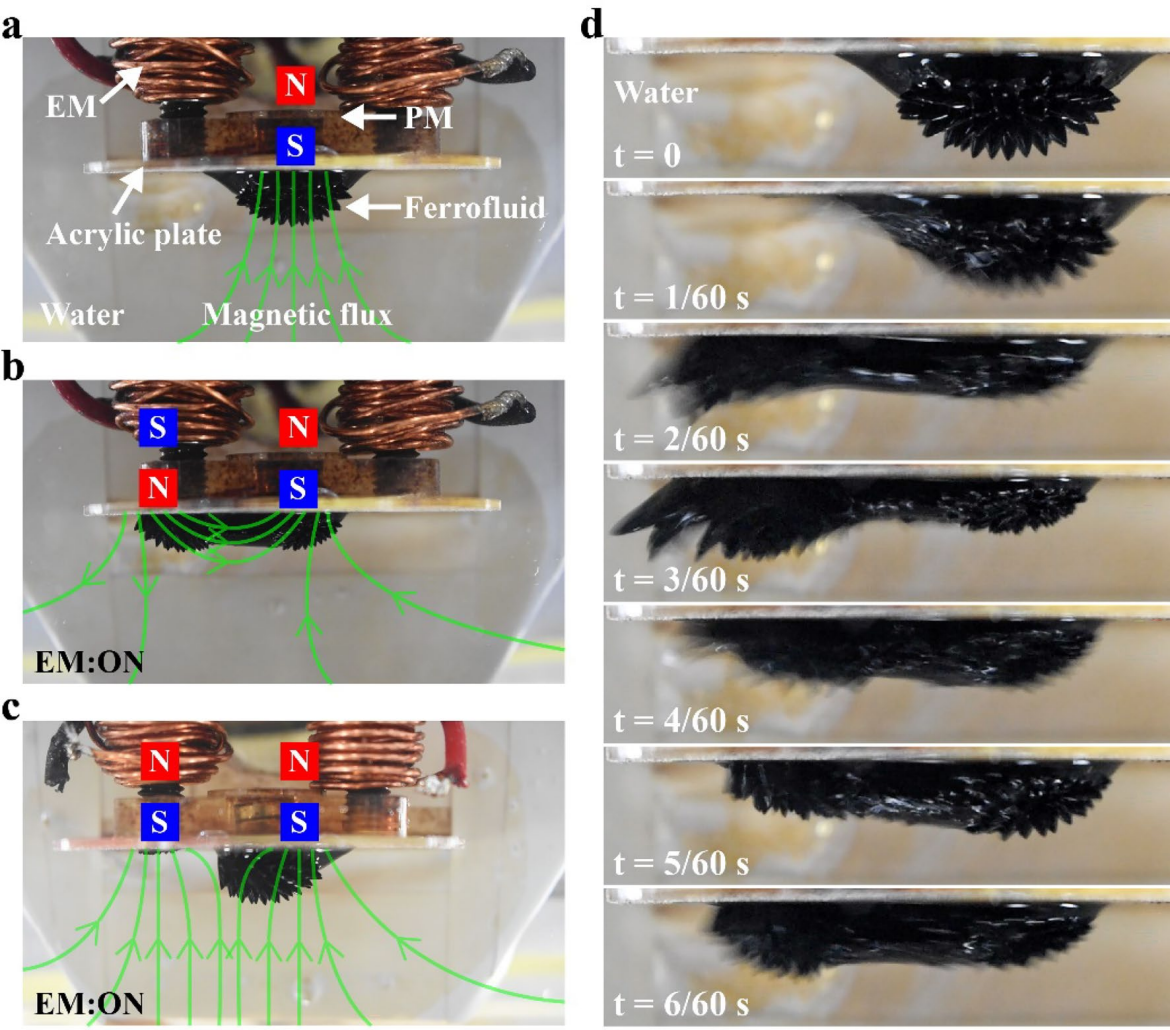
(**a**) Ferrofluid suspended on an acrylic plate by a permanent magnet placed behind. (**b**) Activation of the electromagnet using the polarity opposing to the PM causes a large deformation of the ferrofluid. (**c**) Actuation of the EM using the polarity same to the PM results in a small deformation. (**d**) Sequence of the movement of ferrofluid under activation of EM (see also Supplementary Video S1).

A ferrofluid changes its shape in response to an applied magnetic field. The deformation follows the magnetic flux lines that appear as spikes as shown in Fig. 2a, where the ferrofluid is trapped by a permanent magnet (PM) via an acrylic plate. The entire structure is immersed in water. In this state, the fluid remains almost static because the magnetic flux is constant. In the figure, N and S indicate the polarity of the magnet. When another magnetic field having opposite polarity is applied by an electromagnet (EM), the shape of ferrofluid is changed, as displayed in Fig. 2b. The fashion of shape change depends on the intensity and polarity of the applied magnetic fields. When the polarity of the magnetic fields is the same, the shape change of the ferrofluid reduces, as depicted in Fig. 2c. The reason for this is that the magnetic flux lines of the two magnets push each other away and the ferrofluid deforms along them. We refer to deformation resulting from opposing polarities as “large deformation” and that generated from the same polarities as “small deformation”.

The speed of shape change of ferrofluid in the water is ~ 0.1 s, as can be seen from the sequence of large deformation represented in Fig. 2d (see also Supplementary Video S1). This sequence is taken at 60 fps, so the time step is 1/60 s. When the EM is activated, the ferrofluid rapidly moves towards the newly generated magnetic flux. The deformation is the largest at 3/60 s. After that, the ferrofluid slightly recedes because of the surface tension that tries to keep the shape in a minimized state. This stabilization process continuous until 6/60 s. Supplementary Video S1 is available for the deformation process explained above. The rapid movement of the ferrofluid during the large deformation is expected to generate reaction forces by pushing the surrounding water, which can be exploited as a thrust force to move the robot. In the case of the small deformation, the force is smaller. Reaction forces may also arise from the friction between the ferrofluid and water.

**Figure 3:**
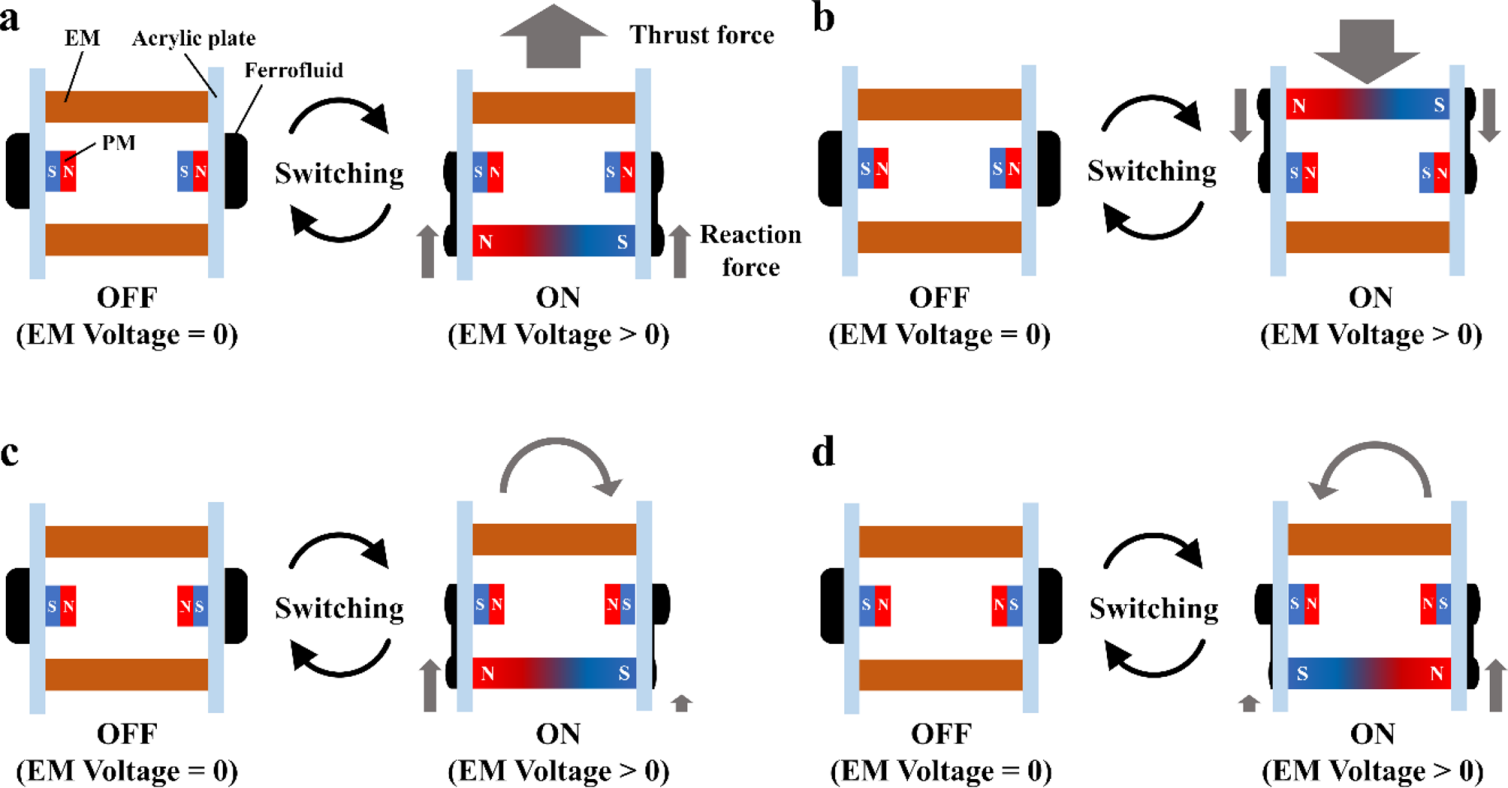
Locomotion principle of the robot. Inducing a large deformation of ferrofluid on both sides moves the robot (**a**) forward and (**b**) backward. (**c**) Generation of simultaneous large and small deformations causes an asymmetric distribution of reaction forces, leading to rotational movement of the robot. (**d**) The direction of the rotational movement can be inverted by changing the polarity of EM.

Various locomotion patterns can be formed by the combination of large deformations and small deformations. Figure 3a illustrates a simplified schematic of the robot, which consists of EMs and PMs attached to acrylic plates. Ferrofluid is held on the surface of each acrylic plate. As shown in Fig. 3a, when the PMs and EMs are set such that opposite polarities appear on both sides, the activation of one of the EMs results in a large deformation of the two ferrofluids in the same direction. Continuous switching (ON/OFF) of the EM leads to a thrust force pushing the robot in a straight line. Similarly, when the other EM is instead activated, the robot moves in the opposite direction, as shown in Fig. 3b.

Rotational movement can also be achieved by setting up the PMs to have the same polarity on one side and the opposite polarity on the other, as shown in Fig. 3c. In this case, large deformation and small deformation occur simultaneously, causing an asymmetric distribution of the reaction forces that rotates the robot. The direction of the rotation can be inverted by changing the polarity of the EM, as depicted in Fig. 3d.

From a systematic point of view, the polarity of EMs can be changed by switching the direction of the electrical current. The polarity of PM, as can be seen in Fig. 3, needs to be physically changed (flipped) between the linear and rotational movements. This requires an additional mechanism, which would make the structure of the robot complicated. However, this issue could be addressed by splitting each EM in two, which enables the generation of various combinations of polarity independently on each side. In order to simplify the characterization of the robot locomotion, we consider only the configurations shown in Fig. 3a and c in the rest of the paper.

**Figure 4:**
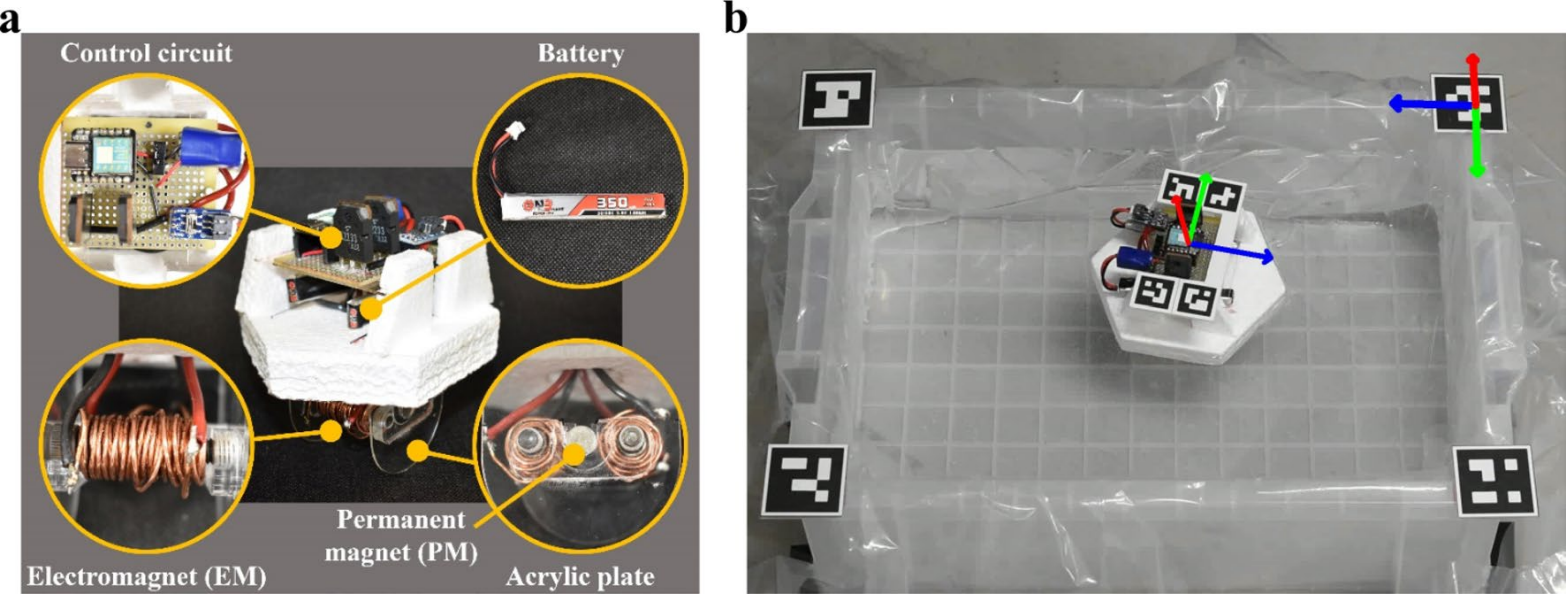
(**a**) Structure of the robot developed in this study. (**b**) The robot and water tank equipped with fiducial markers with coordinates of them overlain. Relative position and direction of the robot to the case was calculated using them.

The robot developed in this study is displayed in Fig. 4a. The fuselage is made of laser-cut styrofoam. On the top is the electrical circuit for control, which is powered by a couple of LiPo batteries (capacity 350 mAh) placed underneath. The circuit mainly consists of a microcontroller (Seeeduino XIAO) and FETs (2SK2233) and inputs electric power to the EMs using a square wave at programmed frequency.

Under the fuselage is the moving unit composed of EMs, PMs, and acrylic plates. This unit is underwater when the robot is placed on the water surface. The EMs are fabricated by coiling a copper wire (diameter 1 mm) onto a hollow steel core (diameter 8 mm and length 25 mm) followed by inserting an M6 steel bolt (length 35 mm). These steel parts are made of chromium–molybdenum steel (SCM435). The number of turns and the electrical resistance of the coil are 63 and 0.45 Ω, respectively. With the control circuit, the EM can generate a magnetic flux density up to 81.8 mT on the surface of the pole and 69.7 mT on the acrylic plate where the ferrofluid is held. These values were acquired using a Tesla meter (TM-801, Kanetec). The distance between the two EMs is 26 mm. The PMs used in this study are neodymium magnets having a diameter and thickness of 8 and 4 mm, respectively. Each of them generates a magnetic flux density up to 380.0 mT on the surface and 219.0 mT on the acrylic plate (measured by the Tesla meter). They are attached to an acrylic plate having a thickness of 1 mm. The planar dimensions of the robot are 107 mm long and 94 mm wide. It has a mass of 127 g without ferrofluid. In the robot, each component, e.g., the control circuit, moving unit, and batteries, is placed to ensure that the center of mass is positioned at the center of the robot, thereby avoiding mass imbalance. This minimizes any potential bias that could occur in the trajectory of the robot.

**Figure 5:**
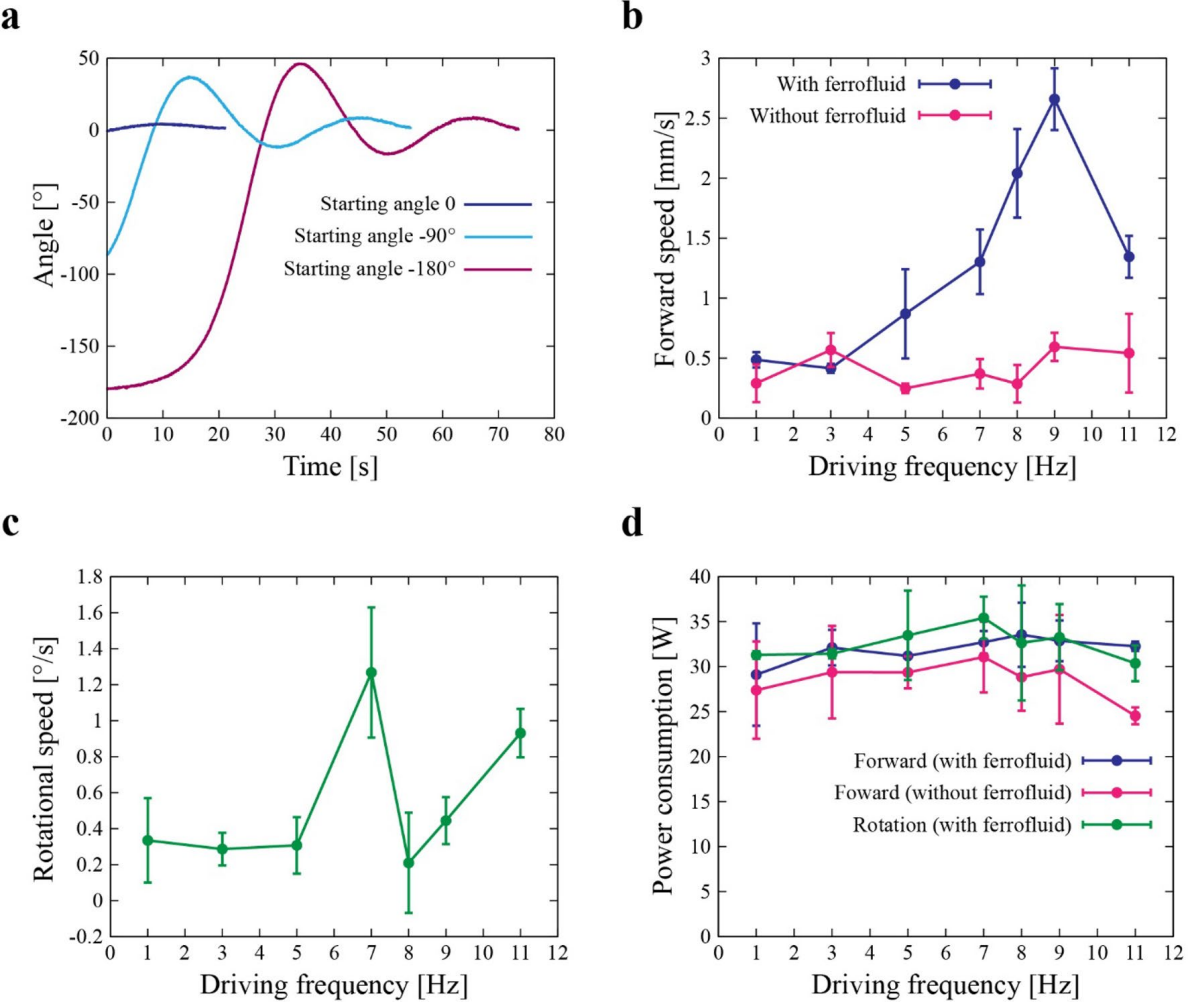
(**a**) Angular displacement of the robot in the inactive state as a function of the time. The angle converges to 0° (relative to the direction of water tank) regardless of the starting angle. (**b**) Measured translational speed as a function of the driving frequency with and without ferrofluid. (**c**) Measured rotational speed as a function of the driving frequency. (**d**) Measured electric power consumption as a function of the driving frequency.

During preliminary tests, we found the robot acts like a compass because of the environmental magnetic fields such as geomagnetism or magnetic field caused by the iron components of the building. Therefore, the direction of the water tank and the robot was set carefully during the characterization to minimize the influence of environmental magnetic fields and to clearly visualize the locomotion by fluid–fluid interaction. To determine the suitable initial condition for the characterizations of the robot, we set the permanent magnets of the robot as Fig. 3a, put it quietly in different initial directions, and observed the motions while keeping the robot turned off. The coordinate of the robot was tracked using the markers as shown in Fig. 4b (see “Method” section for more detail). As can be seen in the Fig. 5a, the direction of the robot converges to around 0° (which is relative to the direction of water tank) when the EMs are not activated. Therefore, we set the initial angle of the robot to 0° in every experiment.

**Figure 6:**
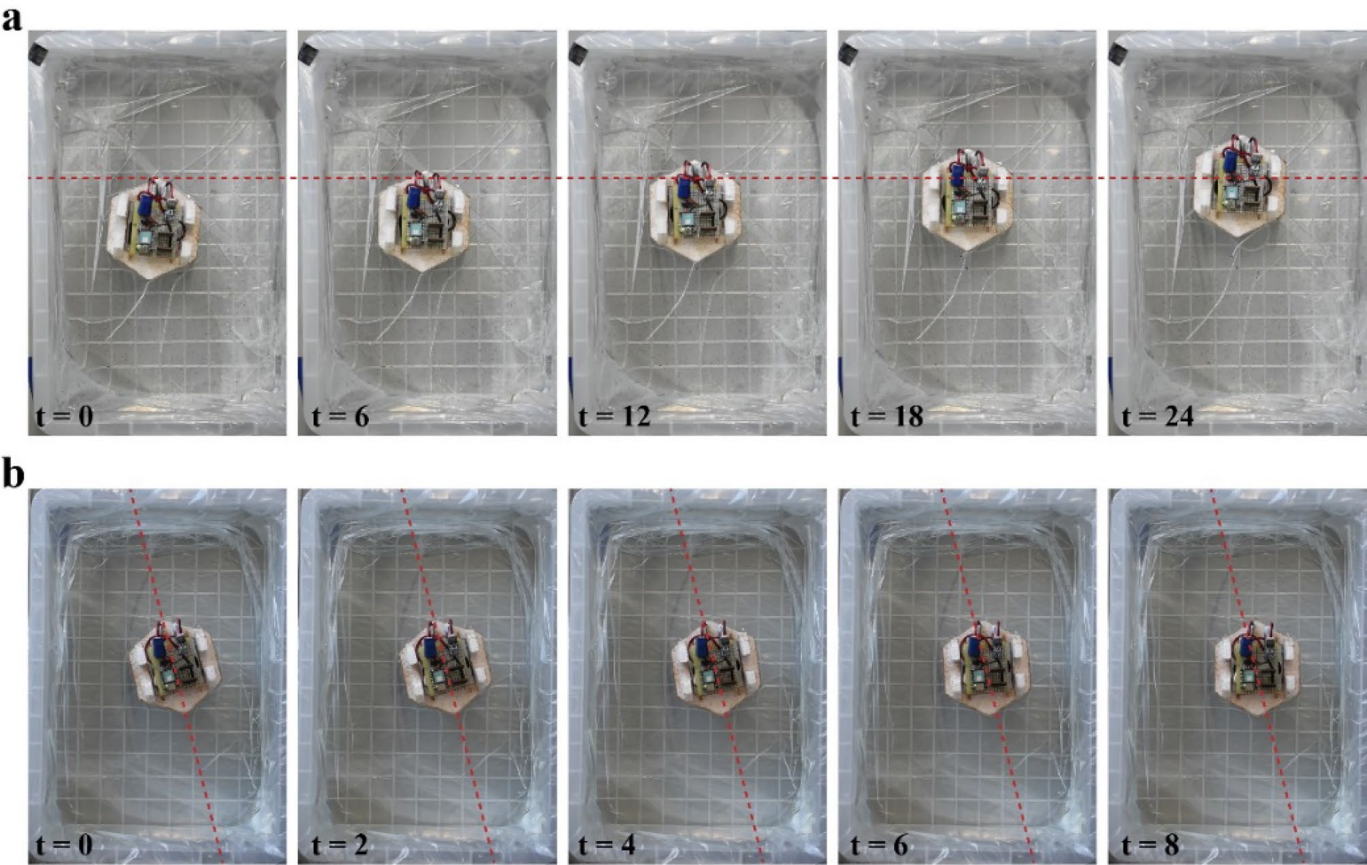
(**a**) Forward movement and (**b**) rotational movement of the robot (driving frequency 3 Hz).

After setting up the initial condition, we observed a forward and rotational movement as expected from the working principle previously discussed. The sequence of these movements is displayed in Fig. 6. Activating the EMs generates the movements according to various driving modes. Supplementary Video S2 is available for the locomotion of the robot.

We then quantified the speed of the robot (see “Method” section for more detail). The measured forward speed as a function of the driving frequency is plotted in Fig. 5b. It takes the peak velocity as 2.7 mm/s at 9 Hz. This may result from the dynamic characteristics of the ferrofluid, i.e., the deformation of fluid may be under resonance and the largest in the tested frequency range. While the speed of the robot without the ferrofluid is steady, the speed of the robot with ferrofluid become increased from 5 Hz and reached the top at the 9 Hz. Interestingly, the direction of the translation was reversed from the driving frequency 9 Hz. This can be a result of dynamic characteristics of the ferrofluid. As shown in Fig. 2d, it takes ~ 0.1 s for ferrofluid to deform from the EM is turned on. Therefore, if driven at high frequency, ferrofluid would not have enough time to snap back to the original shape while the EM is turned off. In such case, ferrofluid probably stay near the EM to changes flow of ferrofluid and reverse the direction to proceed. In the measured data, the amount of speed is clearly different between the cases with and without ferrofluid, demonstrating the effect of fluid–fluid interaction for generating robot movements.

There are two possible reasons associated with the locomotion without ferrofluid (up to ~ 0.5 mm/s). The first reason is the presence of Lorentz force generated in the robot. As seen in Fig. 4a, the electrical terminals to the coil of the EM are exposed, meaning that they are in contact with the surrounding water. When a voltage is applied, the majority of the electric current flows through the coil, while a smaller current also passes between the terminals through the water. The resultant Lorentz force between the magnetic field generated by the electric current passing through the coil and the small current flowing through the water may have pushed the robot. Accordingly, insulating the terminals can minimize the influence of Lorentz force. The second reason is the presence of magnetic fields generated by induced electric currents around the robot. The active change in the magnetic flux of the EM generates tiny induced currents in the surrounding water. The magnetic fields generated by these induced currents interact with the magnetic field of the robot, potentially resulting in reaction forces. In the conditions tested in this study, the rear EM relative to the direction of locomotion is activated, causing the reaction forces to push the robot forward.

The measured rotational speed as a function of the driving frequency is plotted in Fig. 5c. The rotational speed increases with the frequency and becomes 1.2°/s at 7 Hz. This trend is different from the case of the forward speed. In addition, rotational direction was also reversed from driving frequency 9 Hz and the direction changed time to time in with 8 Hz. This can be caused by the dynamic characteristics of ferrofluid changed around 8 Hz. The influence of environmental magnetic fields, which probably exert a restoring force to return the robot to a certain direction, may be responsible. This may also be the cause of errors appearing in both measurements. Considering the characteristics in the driving frequency 8 Hz where translation direction was the same to the lower frequency cases and, rotation direction was unstable, the different way of physical oscillation of the robot centroid caused by the motion of the ferrofluid in the translation and rotation mode (Fig. 3a,c) would result in the different dynamic characteristics of the robot.

Peak frequencies observed in the forward and rotational movements are 9 Hz and 7 Hz, respectively. As mentioned previously, these peak frequencies suggest a resonance mode that increases the deformation of the ferrofluid. The difference in peak frequency values for forward and rotational motions may be attributed to the relative speed of the surrounding water and the ferrofluid. At the peak, the forward speed is 2.7 mm/s and the rotational speed is 1.1 mm/s on the side of the robot with the ferrofluid (calculated from the rotational speed of 1.2°/s with half of the robot length of 107 mm as radius). We assume that water inhibits the ferrofluid's deformation by acting as a load on it. Due to the direction of deformation in which the ferrofluid generates thrust and the direction of water flow alignment, in the resonant state, the ferrofluid faces a smaller load at the forward movement and, conversely, a larger load at the rotational movement, which is thought to have caused the resonant frequency to be higher in the former case and lower in the latter.

We next measured electric power consumption as a function of the driving frequency as displayed in Fig. 5d. Depending on the type of movements and the frequency, the robot consumes roughly 30–38 W. There is a clear difference between the cases with and without ferrofluid. The case with ferrofluid consumes more power, suggesting that the electric energy is transferred to the deformations of ferrofluid therefore the kinetic movements of the robot.

**Figure 7:**
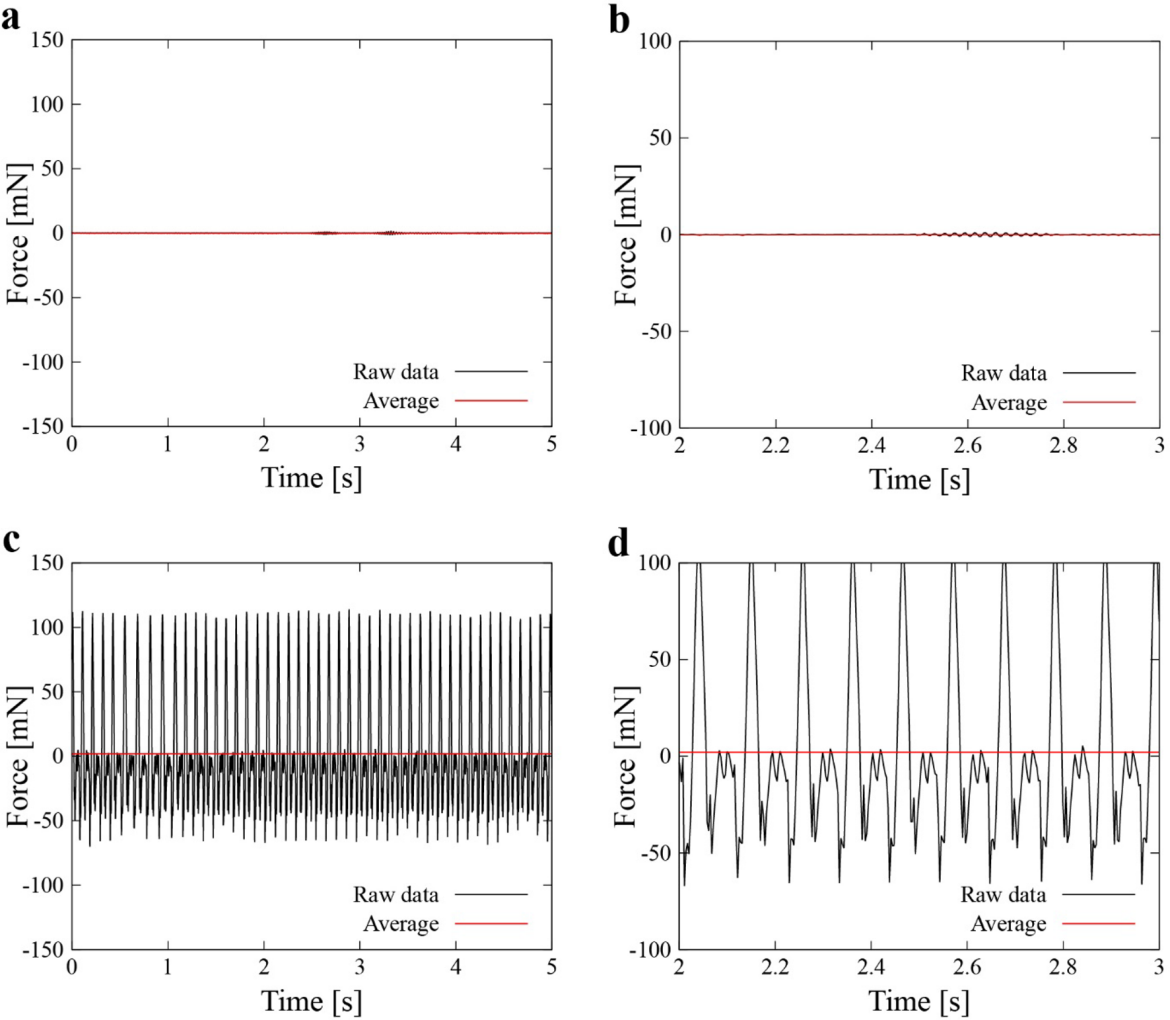
Measured force of the robot as function of the time. (**a**, **b**) Measured force when the robot is inactive. (**c**, **d**) measured force when the robot is active.

We further quantified the movements of the robot to support that the movements are consequence of fluid–fluid interaction. To do so we looked at force acting on the robot, with hypothesis that if robot moves the net force is not zero and vice versa. Figure 7 shows the thrust force measured by a low capacity, high precision load cell (see “Method” section for more detail). In this figure, the force value exhibits asymmetric trend due to the interaction between the ferrofluid and surrounding water, and the average force values is above zero. The measured net thrust force was found to be 2 mN from which it is clear that the movements of the robot was caused by fluid–fluid interaction. Overall, the experimental results illustrate the successful implementation of our principle.

## Concluding remarks

We have demonstrated robot locomotion by fluid–fluid interaction. The robot developed in this study employs ferrofluid, whose active deformations generate movements in water. During the experiments, we observed the influence of environmental magnetic fields on the movements of the robot. The presence of these external fields may cause an error in the measured data. To mitigate this issue, magnetic shielding will be implemented in the experimental setup, and further characterization of the robot will be carried out. A variety of locomotion patterns, a wider range of driving frequency, and force are tested. Through these experiments, the characteristics of the robot will be clearly determined.

Investigation of liquid combinations for locomotion of fluid–fluid interaction remains an aspect of future work. The current study employs water as the surrounding fluid, but other liquids such as seawater and blood can be considered in the future. In addition, the robot body could also be changed from ferrofluid to liquid metals. To use different liquids as the surrounding fluid, the effects of their electrical conductivity and magnetic permeability on locomotion should be investigated carefully. As the experimental results suggest, the conductivity may contribute to the generation of induced currents; therefore, the induced magnetic fields in the surrounding liquid due to the magnetic field generated by the robot ultimately affect the way it moves. In a liquid with high magnetic permeability, the magnetized liquid may cling around the ferrofluid, which may inhibit deformation when the EMs are active. However, the collision of the moved ferrofluid with the magnetized liquid attracted by the EM could generate a large reaction force, which may conversely increase the locomotion speed of the robot. Modeling of fluid–fluid interaction should also be conducted. It is important to reveal the mechanism and condition of proceeding direction reverse to occur which can be utilized for more rational hardware setup and controller. For this purpose, computational fluid dynamics coupled with electromagnetism is a powerful tool. The outcome of these works will contribute to define the application of fluid–fluid robots.

As suggested in the experimental result discussed above, locomotion efficiency may be an important issue. One potential approach to address this issue is to investigate optimal design parameters (e.g., intensity of magnetic field, amount of ferrofluid, and geometry of magnets) to maximize the locomotion performance through experimental and computational environments. Especially, electromagnets in the experiments consume roughly 10 A of current. Replacing the electromagnets to one with more thin and dense wire would be effective.

Based on the results of the aforementioned research efforts, fluid–fluid robots of diverse configurations and size scales could be designed for various applications. We are interested in the configuration of robots that are completely covered with ferrofluid or other liquids able to function as the body. This type of robot would exhibit a wide range of locomotion patterns and act as a cell of modular robots. Potential applications of fluid–fluid robots include inspection, delivery, and sampling in fluid environments, such as rivers, seas, and inside the human body. In these environments, the liquid nature of the robots assures the safety of the surrounding objects. This is an expected advantage of robotic systems that locomote based on fluid–fluid interaction.

## Methods

Measurement and subsequent calculation of the translation and angular displacement was carried out with the fiducial markers on the robot and the corners of the water tank (Fig. 4b). These groups of markers respectively construct robot coordinate and water tank coordinate so that relative translation and rotation of the robot can be extracted from the movie taken by a camera (D3500, Nikon) using OpenCV. The relative position of the robot coordinate measured by the water tank coordinate as a robot position was employed, while angular displacement between the x-axes of these coordinates in the xy-plane of the water tank coordinate was used for determining the rotational motion of the robot. Since the movies were taken from almost right above the water tank, z-axes of both estimated coordinates were unstable while others were stable. However, this seemed to make little error for the result, because robot moved and rotated in the xy-plane of these coordinates. Note that our EMs are so stronger than PMs that response of the robot for the environmental magnetic field was determined by the polarity of the EMs when the robot turned on. Therefore, appropriate initial direction of the robot considering the polarity of the EMs was applied in all the experiments.

The locomotion of the robot in terms of forward speed and rotational speed was characterized as follows. 1.0 g of ferrofluid (DS-60, Sigma Hi-Chemical) was put on each acrylic plate on both sides. The ferrofluid consists of isoparaffin (40–60%) and triiron tetraoxide (40–60%). Its physical properties include a specific gravity ranging from 1.3 to 1.5, a typical viscosity of 50 to 150 mPa s, and a saturation magnetization typically between 40 and 50 mT (measured at 20 °C). The robot was then placed on the water surface in a tank (475 mm × 250 mm × 140 mm) equipped with fiducial markers on each the corners. The moving unit of the robot was activated, and subsequent locomotion was captured using the camera. The recorded video was then processed to extract the moving speed of the robot. The forward speed was calculated by dividing the travel distance by the measurement time of 20 s. To prove that the translational speed was caused by the ferrofluid, the translational speed using the robot without the ferrofluid was also measured. The rotation speed was calculated by dividing the maximum angular displacement by the time to reach that angle. During the experiment, the applied voltage to the EMs was set to 4.35 V, which induces an electric current of ~ 9.5 A. The driving frequency were 1, 3, 5, 7, 8, 9, and 11 Hz. At each frequency, three measurements were taken, and their average and standard deviation were reported. After each measurement, the electric power consumed was calculated using a battery charger (iMAX b6, SkyRC).

The thrust force generated by the ferrofluid was measured with a low capacity, high precision force sensor (FS1M-1NB, THK-Precision), signal amplifier (FSA201C, THK-Precision), and multimeter (2100/100, Keithley). A fixture made of 3D printer was used to connect the sensor to the robot directly. An additional float was added to the robot to offset the mass of the fixture (~ 50 g). The thrust force was measured with the translational mode (Fig. 3a) and driving frequency at 9 Hz, which achieved the highest translational speed in the experiment. Since the output voltage of the amplifier was linear to the applied force, data sequence of the voltage was firstly recorded with the inactive robot, and then activated the robot. The average value of forces of both active and inactive state using 5-s-long data sequence each. The difference of the two averaged forces represents the thrust force.

### Supplementary Information

Supplementary Video S1.

Supplementary Video S2.

## Data Availability

All data that support the plot within this paper and other findings of this study are available from the corresponding author upon a reasonable request.
